# Comparative Analysis of Vitamin D, Folic Acid, and Trace Minerals Levels in Various Stages of Invasive Ductal Breast Cancer

**DOI:** 10.7759/cureus.70593

**Published:** 2024-10-01

**Authors:** Vemuri Helena, Suresh Arumugam, Natrajan Muninathan, Kuppusamy Baskaran, Amena Tasneem

**Affiliations:** 1 Biochemistry, Meenakshi Medical College Hospital and Research Institute, Meenakshi Academy of Higher Education and Research, Kanchipuram, IND; 2 Central Research Laboratory, Meenakshi Medical College Hospital and Research Institute, Meenakshi Academy of Higher Education and Research, Kanchipuram, IND; 3 Biochemistry, Shadan Institute of Medical Sciences, Hyderabad, IND

**Keywords:** antioxidant minerals, cancer progression, copper, immune function, invasive ductal breast cancer, magnesium, nutrient levels, vitamin d, zinc

## Abstract

Background: Invasive ductal carcinoma (IDC) is one of the most commonly diagnosed subtypes of breast cancer, representing the majority of breast cancer cases. This study investigates the levels of vitamin D, folic acid, and antioxidant minerals (zinc (Zn), copper (Cu), and magnesium (Mg)) in IDC patients across different disease stages to explore their potential roles in disease progression.

Methods: We analyzed a cohort of 150 female patients with IDC, aged between 30 and 67 years (51 ± 15.5 years). Blood samples were collected to measure levels of vitamin D, folic acid, Cu, Mg, and Zn. Patients were categorized into Stage 1 to Stage 4 of cancer. Variations in nutrient levels across these stages were statistically assessed using ANOVA and post-hoc tests.

Results: The study results revealed varying levels of key nutrients across different stages of the disease. Vitamin D levels averaged 17.7 ng/mL, with higher concentrations in early-stage patients, indicating a possible correlation with disease severity. Folic acid levels had a mean of 12.2 ng/mL, showing a decline in later stages, potentially linking it to cancer progression. Copper levels averaged 161.3μg/dL, peaking at 170μg/dL in Stage 3, suggesting a connection with cancer aggressiveness. Magnesium levels, with an average of 1.4 mg/dL, dropped notably to 0.6 mg/dL by Stage 4, highlighting its possible involvement in disease progression. Zinc levels averaged 69.4 μg/dL, with a significant decrease in advanced stages, emphasizing its importance for immune function and cellular health.

Conclusion: The study demonstrates significant variations in the levels of vitamin D, folic acid, and antioxidant minerals across different stages of IDC. These nutrients may influence cancer progression, underscoring the potential benefits of nutritional assessments and interventions in managing IDC.

## Introduction

Invasive ductal breast cancer (IDC) is the most common subtype of breast cancer, accounting for approximately 80% of all breast cancer cases [1, 2]. A complex interplay of biological and environmental factors influences the progression and prognosis of IDC, with nutritional status emerging as a key component [3]. Essential nutrients, including vitamin D and antioxidant minerals such as zinc (Zn), copper (Cu), and magnesium (Mg), play critical roles in maintaining cellular health and immune function, both of which are vital in the prevention and management of cancer [4-6].

Vitamin D is well recognized for its benefits to bone health, yet recent studies have also highlighted its considerable anticancer properties [7-9]. Sufficient levels of vitamin D can play a crucial role in inhibiting the growth and proliferation of cancer cells. Vitamin D has been shown to regulate cellular differentiation, helping cells to maintain their normal structure and function, which may prevent abnormal or cancerous growth. Additionally, vitamin D enhances the immune system's response, potentially increasing the body’s ability to detect and eliminate cancer cells, thereby contributing to overall cancer prevention and immune regulation [10-13]. Folic acid, crucial for DNA synthesis and repair, may also influence cancer progression through its roles in cellular proliferation and differentiation [14, 15].

Antioxidant minerals like Zn, Cu, and Mg are essential for a range of physiological functions. Zinc supports immune function and cellular metabolism, Cu contributes to angiogenesis and tumor growth [16], and Mg is indispensable for numerous enzymatic reactions and metabolic processes [17]. Deficiencies or imbalances in these nutrients can potentially accelerate cancer progression and adversely affect patient outcomes.

Although these nutrients play vital roles in cellular function and immune defense, there is a noticeable scarcity of data regarding their levels across different stages of IDC. This study aims to address this gap by thoroughly investigating the concentrations of vitamin D, folic acid, and key antioxidant minerals in patients with IDC at various stages of the disease. The primary hypothesis of this research is that nutrient levels may fluctuate with disease progression, potentially influencing cancer growth and patient outcomes. By examining these relationships, this study seeks to emphasize the critical role that nutritional assessments and targeted interventions could play in improving the management, treatment strategies, and overall prognosis of IDC.

## Materials and methods

Study design and setting

This cross-sectional study was conducted at Shadan Institute of Medical Sciences, Hyderabad, Telangana, India, spanning from July 2022 to May 2024.

Study population

The cohort comprised 150 female patients diagnosed with IDC. These patients were stratified into Stage 1 to Stage 4 of the disease, based on clinical evaluations and established diagnostic criteria.

Inclusion and exclusion criteria

Inclusion Criteria

This study required female participants between the ages of 30 and 67 years who had a confirmed diagnosis of IDC. This is most commonly diagnosed in women within this age range, covering both premenopausal and postmenopausal patients, which allows for a comprehensive understanding of how nutrient levels may vary across different age groups. This range also reflects the typical onset and progression of breast cancer, ensuring that our study captures relevant variations in disease stages and patient outcomes. Additionally, selecting this age group helps exclude very young or much older patients, who might have different biological factors or comorbidities that could confound the results. Only those individuals with a definitive histopathological diagnosis of IDC were considered eligible for the study, allowing us to focus on this specific subtype of breast cancer for accurate and relevant analysis.

Exclusion Criteria

Patients who had been diagnosed with other forms of breast cancer, apart from IDC, were excluded from this study to maintain a clear focus on IDC-specific findings. Additionally, individuals who were currently receiving treatment with nutritional supplements were not included, as this could interfere with the accurate measurement of natural nutrient levels in the body. Furthermore, patients with known metabolic or autoimmune disorders were excluded to avoid potential confounding factors as these conditions could independently affect nutrient metabolism and immune function, skewing the study’s results.

Data collection

Demographic data, including age and cancer stage, were systematically recorded. Blood samples were collected from each participant to measure levels of vitamin D, folic acid, Cu, Mg, and Zn. 

The samples were collected and analyzed in batches rather than one at a time. During the collection process, all samples were properly stored under controlled conditions to maintain their integrity. Specifically, they were preserved at appropriate temperatures (as per standard guidelines for each type of sample) until analysis could be performed.

Regarding the impact of long-term storage, we followed established protocols to ensure that any potential degradation or alteration of the samples was minimized. While extended storage can theoretically affect certain biomarkers, the preservation methods we used were designed to prevent such changes. Additionally, we adhered to standardized time frames for analysis, ensuring that any potential variability in nutrient levels due to storage was avoided.

Biochemical analysis

Vitamin D Levels

Vitamin D levels were assessed using the enzyme-linked immunosorbent assay (ELISA) technique, which was conducted with the aid of an automated chemiluminescence analyzer, model PAT700. This highly sensitive and specific method allowed for precise detection of Vitamin D concentrations in the samples. The results were quantified in nanograms per milliliter (ng/mL), providing accurate and standardized measurements for analysis [18-19].

Folic Acid Levels

The levels were determined by ELISA, which was conducted with the aid of an automated chemiluminescence analyzer, model PAT700. Results were reported in ng/mL [18-19].

Copper and Zinc Levels

Stored serum samples were brought to room temperature and subsequently diluted with 1% nitric acid at a 1:5 ratio (1 mL of serum mixed with 4 mL of 1% nitric acid) to facilitate deproteinization. The Cu and Zn concentrations in the serum were then measured using atomic absorption spectroscopy (AAS), performed on the Electronic Corporation of India Limited (Hyderabad, India) model 492.

The AAS technique is based on the principle that metal atoms strongly absorb light at distinct characteristic wavelengths, which correspond to the emission spectral lines of the specific element. For the measurement of serum Cu and Zn, hollow cathode lamps for each element were utilized. The hollow cathode lamp emits a spectrum of light that passes through the flame and is focused on the entrance slit of a monochromator, which is adjusted to capture the intensity of the specific spectral line. The light at this wavelength is absorbed by the flame.

For the elemental analysis, working standards with concentrations of 0, 0.25, 0.5, 0.75, and 1 μg/mL for both Cu and Zn were prepared. By measuring the absorbance of these working standards with known concentrations, the concentrations of Cu and Zn in the serum samples were determined. The absorbance for Cu was measured at a wavelength of 324.8 nm, while for Zn, it was measured at 213.9 nm. The final values were represented in μg/dL [20].

Magnesium Levels

Magnesium levels were measured using colorimetric methods, with results expressed in mg/dL [21].

Statistical analysis

Data analysis was conducted using IBM SPSS Statistics software, version 24 (IBM Corp., Armonk, NY). Descriptive statistics, including means and standard deviations, were calculated for each nutrient. Stage-related differences in IDC were examined through ANOVA, with post-hoc tests applied to identify significant disparities between stages. Statistical significance was set at a p-value below 0.05. The normality of each marker, including Zn levels, was tested using the Shapiro-Wilk test. For non-normally distributed data, non-parametric tests such as the Kruskal-Wallis test were utilized, while ANOVA was used for data meeting normality assumptions to assess group differences.

Ethical considerations

Ethical approval was granted by the Institutional Ethics Committee of Shadan Institute of Medical Sciences (approval number 012/SIMS/Research/2022). Written informed consent was obtained from all participants prior to their inclusion in the study.

## Results

Patient demographics

A total of 206 patients were initially screened for eligibility. Of these, 56 were excluded based on predefined criteria, including diagnoses of other forms of breast cancer, current use of nutritional supplements, or the presence of metabolic or autoimmune disorders. After applying these exclusion criteria, we finalized a sample size of 150 eligible participants. These patients, all diagnosed with invasive ductal breast cancer, were classified according to different stages of the disease. The participants' ages ranged from 30 to 67 years, with a mean age of approximately 51 years and SD of 15.5 years. 

Vitamin D levels

Vitamin D levels in the patients varied significantly. The mean vitamin D level was 17.7 ng/mL, with a standard deviation of 7.8 ng/mL. The normal vitamin D levels as per the Food Safety and Standards Authority of India (FSSAI) in Indian adults are 20-30 ng/mL. Patients in the early stages (Stage 1) had slightly higher vitamin D levels, while those in later stages (Stage 4) showed a marked decrease. This suggests a potential link between disease progression and vitamin D deficiency (Table 1).

**Table 1:** Vitamin D and folic acid levels across different stages of invasive ductal carcinoma

Stage	Vitamin D (ng/mL) Mean ± SD	Folic acid (ng/mL) Mean ± SD
All	17.7 ± 7.8	12.2 ± 6.7
Stage 1	19.8 ± 7.0	12.1 ± 5.7
Stage 2	17.4 ± 3.7	13.8 ± 5.0
Stage 3	17.1 ± 5.7	12.9 ± 6.1
Stage 4	8.0 ± 0.0	1.1 ± 0.0

Folic acid levels

Folic acid levels among the patients also showed variation. The mean folic acid level was 12.2 ng/mL, with a standard deviation of 6.7 ng/mL. The normal folic acid levels as per FSSAI in Indian adults are 2.7-17.0 ng/mL. Patients in the later stages of the disease (Stage 3 and Stage 4) exhibited lower folic acid levels compared to those in the early stages (Table 1).

This decrease in folic acid levels may indicate its potential role in disease progression and the importance of maintaining adequate levels for better health outcomes.

Copper levels

Copper levels in the study population had a mean value of 161.3 μg/dL, with a standard deviation of 22.5 μg/dL. Patients in Stage 3 had the highest Cu levels, averaging around 170 μg/dL, while Stage 1 patients had lower levels, averaging around 144 μg/dL (Table 2).

**Table 2:** Copper and zinc levels across different stages of invasive ductal carcinoma

Stage	Copper (μg/dL) Mean ± SD	Zinc (μg/dL) Mean ± SD
All	161.3 ± 22.5	69.4 ± 50.7
Stage 1	159.2 ± 21.7	65.9 ± 51.7
Stage 2	165.8 ± 10.8	75.7 ± 46.6
Stage 3	170.0 ± 25.9	56.2 ± 62.2
Stage 4	165.0 ± 0.0	44.0 ± 0.0

The increased Cu levels in more advanced stages may suggest a correlation with the aggressiveness of the cancer.

Zinc levels

Zinc levels varied widely among the patients with a mean of 69.4 μg/dL and a standard deviation of 50.7 μg/dL. Stage 1 patients had higher Zn levels, averaging around 118 μg/dL, whereas Stage 4 patients had much lower levels, averaging around 36 μg/dL (Table 2).

This significant drop in zinc levels in advanced stages may reflect its role in immune function and cellular health, highlighting the need for adequate zinc intake for cancer patients.

Magnesium levels

Magnesium levels across the patient cohort had a mean of 1.4 mg/dL and a standard deviation of 0.8 mg/dL. Patients in Stage 1 exhibited higher magnesium levels (mean of 2.1 mg/dL), while those in Stage 4 had significantly lower levels (mean of 0.6 mg/dL) (Table 3).

**Table 3:** Magnesium levels across different stages of invasive ductal carcinoma

Stage	Magnesium (mg/dL) Mean ± SD
All	1.4 ± 0.8
Stage 1	2.1 ± 1.0
Stage 2	1.2 ± 1.0
Stage 3	1.0 ± 0.8
Stage 4	0.6 ± 0.0

The decline in magnesium levels with advancing cancer stages could indicate its potential involvement in cancer progression and the body's metabolic response to the disease.

## Discussion

This study aimed to assess the levels of vitamin D, folic acid, and antioxidant minerals (Zn, Cu, and Mg) in patients with IDC across various disease stages.

The mean vitamin D level in the study population was 17.7 ng/mL, which falls below the optimal range, indicating a widespread deficiency among patients with IDC. Interestingly, vitamin D levels were higher in patients in the early stages of the disease (Stage 1) and progressively declined as the disease advanced, reaching their lowest in Stage 4. This trend aligns with existing literature, which suggests that lower vitamin D levels are often associated with more advanced stages of cancer and are linked to poorer clinical outcomes. Vitamin D involvement in cell differentiation, apoptosis, and immune modulation may underpin its protective effects against cancer progression [18]. Enhancing vitamin D intake through diet, supplements, or sun exposure could therefore support better outcomes in IDC patients [19].

Folic acid levels exhibited a decline as the cancer progressed, with a mean concentration of 12.2 ng/mL. This reduction was especially pronounced in the later stages (Stage 3 and Stage 4), a pattern that aligns with findings from previous studies [20-21]. Folic acid is an important part of making and fixing DNA, so not having enough of it could make genomic instability worse and raise the risk of cancer. This shows how important it is to get enough folic acid through food or supplements to possibly slow the progression of cancer [21].

Copper levels showed a notable increase as the disease progressed, reaching their highest concentration in Stage 3 patients, with an average of 170 μg/dL. This elevation in Cu levels is closely associated with tumor growth and metastasis, as Cu plays a crucial role in promoting angiogenesis (the formation of new blood vessels) which is essential for supplying nutrients to the growing tumor. Additionally, Cu facilitates cellular proliferation, further contributing to the advancement of cancer. These findings align with the established link between elevated Cu levels and the aggressiveness of tumor development and spread. This suggests that Cu could serve as a potential biomarker for indicating the aggressiveness of the cancer [22-23]. 

There was a marked decline in Mg levels as cancer advanced, with Stage 1 patients displaying a mean of 2.1 mg/dL and Stage 4 patients showing significantly lower levels at 0.6 mg/dL (P<0.05). Magnesium plays a well-established role in essential biochemical processes, including DNA repair and cellular metabolism. Adequate Mg levels are crucial for maintaining the integrity of these functions. A deficiency in magnesium could impair these critical processes, leading to cellular dysfunction, which may contribute to the progression of cancer by compromising the body's ability to regulate cell growth and repair damaged DNA [23-24]. This finding supports the potential benefit of Mg supplementation, especially for patients in advanced stages of IDC [25-26].

Zinc levels also varied significantly (P<0.001), being highest in Stage 1 (mean: 118 μg/dL) and lowest in Stage 4 (mean: 36 μg/dL). Zinc is essential for immune function and cellular health; its deficiency might weaken immune defense against cancer cells [27]. The substantial drop in Zn levels with advancing IDC stages underscores the importance of maintaining adequate Zn intake to preserve immune function and potentially enhance cancer prognosis [28-30].

Our findings reveal significant variations in nutrient levels that correlate with the progression of IDC, suggesting a potential role for these nutrients in cancer development and progression. These results highlight the importance of nutritional status in the progression of IDC. Proactively managing nutrient deficiencies through dietary modifications or supplementation could become an essential component of comprehensive cancer care. Future research should focus on longitudinal studies to better establish causality and explore the therapeutic impact of addressing these deficiencies in IDC patients.

Limitations

This study's reliance on single data point entries for certain stages due to the nature of our sample collection limits the robustness of statistical comparisons and the generalizability of our findings for those stages. This should be noted as a significant limitation. Furthermore, the cross-sectional design of this research inhibits our ability to draw causal inferences between nutrient levels and the progression of invasive ductal breast cancer. Influential variables such as dietary intake, lifestyle habits, and treatment regimens, which could significantly impact nutrient levels, were not accounted for. To validate and expand upon our findings, further research is necessary. Future studies should engage larger and more diverse populations and consider employing longitudinal designs to explore the dynamics of nutrient levels over time and their direct impact on cancer progression.

## Conclusions

Our study reveals differences in vitamin D, folic acid, and trace minerals (Zn and Mg) across different stages of IDC, demonstrating a clear pattern of nutrient depletion as the disease progresses. Specifically, we observed that vitamin D and magnesium levels were notably lower in the later stages, while Zn levels also declined significantly in the advanced stages. These trends suggest a potential role for these micronutrients in influencing the progression and severity of IDC.

Copper levels, which peaked in Stage 3, may indicate a link between elevated Cu and the aggressiveness of the cancer. In contrast, higher nutrient levels were seen in the early stages, pointing to a relatively better nutritional status at the onset of the disease or in less advanced cancer. The progressive decline in these nutrient levels in later stages could reflect the increased metabolic demands of the cancer, along with the body's diminished capacity to maintain adequate nutrient levels as the disease advances.

These findings emphasize the importance of integrating regular nutritional assessments into cancer care, as addressing these deficiencies could improve patient outcomes and potentially slow disease progression. Moving forward, our study underscores the need for targeted dietary or supplemental interventions as part of a comprehensive cancer management strategy. Future research should explore the long-term impact of correcting these deficiencies on disease outcomes and survival rates in IDC patients.
